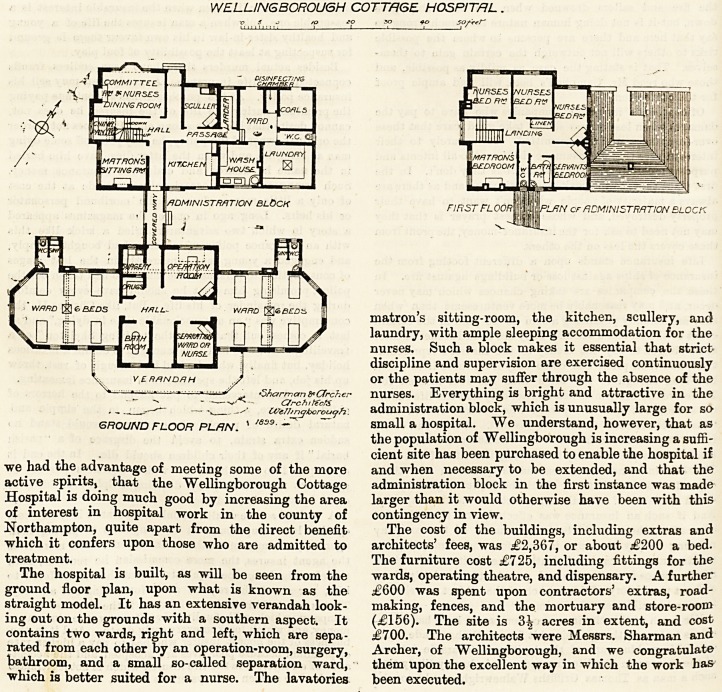# Wellingborough Cottage Hospital

**Published:** 1903-02-07

**Authors:** 


					326 THE HOSPITAL. Feb. 7, 1903.
HOSPITAL ADMINISTRATION.
CONSTRUCTION AND ECONOMICS.
WELLINGBOROUGH COTTAGE HOSPITAL.
We publish to day plans of this institution. It
occupies a commanding site and has ample grounds
surrounding it. It has succeeded in attracting the
interest and support of the well-to-do classes in the
district which it serves, and is well calculated to
meet a felt want by supplying hospital accommoda-
tion to a number of poor persons who formerly had
to travel many miles by railway to secure these
advantages. We have often before dwelt upon the
educational value of cottage hospitals, and we are
inclined to think, from a personal inspection when
we had the advantage of meeting some of the more
active spirits, that the Wellingborough Cottage
Hospital is doing much good by increasing the area
of interest in hospital work in the county of
Northampton, quite apart from the direct benefit
which it confers upon those who are admitted to
treatment.
The hospital is built, as will be seen from the
ground floor plan, upon what is known as the
straight model. It has an extensive verandah look-
ing out on the grounds with a southern aspect. It
contains two wards, right and left, which are sepa-
rated from each other by an operation-room, surgery,
bathroom, and a small so-called separation ward,
which is better suited for a nurse. The lavatories
and w.c.'s are separated from the wards by a cross-
ventilated lobby. The wards themselves are bright
and pleasant in appearance, with abundance of super-
ficial and cubic space. Each has a large bow window
occupying the whole of one end of the ward, which
adds much to their healthiness and attraction. The
furniture and fittings have been carefully selected,
and are, on the whole, good. The drug-room and
dispensary at this cottage hospital embody an excel-
lent idea which has been well carried out. A feature-
of the plan is the administration block, which is con-
nected by a covered way with the hospital proper,
and contains a committee-room, nurses' dining-room,
matron's sitting-room, the kitchen, scullery, and
laundry, with ample sleeping accommodation for the
nurses. Such a block makes it essential that strict
discipline and supervision are exercised continuously
or the patients may suffer through the absence of the
nurses. Everything is bright and attractive in the
administration block, which is unusually large for so
small a hospital. We understand, however, that as
the population of Wellingborough is increasing a suffi-
cient site has been purchased to enable the hospital if
and when necessary to be extended, and that the
administration block in the first instance was made
larger than it would otherwise have been with this
contingency in view.
The cost of the buildings, including extras and
architects' fees, was ?2,367, or about ?200 a bed.
The furniture cost ?725, including fittings for the
wards, operating theatre, and dispensary. A further
?600 was spent upon contractors5 extras, road-
making, fences, and the mortuary and store-room
(?156). The site is 3t> acres in extent, and cost
?700. The architects were Messrs. Sharman and
Archer, of Wellingborough, and we congratulate
them upon the excellent way in which the work has
been executed.
WELLINGBOROUGH COTTAGE HOSPITAL
?c S o to eo 3Q 50f-'re7~~
DISINFECTING
FIRST FLOOfm i SI I PLAN cr ADMINISTRATION BLOCK
matron's sitting-room, the kitchen, scullery, and
laundry, with ample sleeping accommodation for the
nurses. Such a block makes it essential that strict
discipline and supervision are exercised continuously
v ? rrndr h J or the patients may suffer through the absence of the
^   ? ? - ??Sfiarmanfrdrchcr nurses. Everything is bright and attractive in the
uwbnjtoroujh. administration block, which is unusually large for so
ground floor PLfl/v. 1 /es9?~ ~~ small a hospital. We understand, however, that as
the population of Wellingborough is increasing a suffi-
cient site has been purchased to enable the hospital if
we had the advantage of meeting some of the more and when necessary to be extended, and that the
active spirits, that the Wellingborough Cottage administration block in the first instance was made
Hospital is doing much good by increasing the area larger than it would otherwise have been with this
of interest in hospital work in the county of contingency in view.
Northampton, quite apart from the direct benefit The cost of the buildings, including extras and
which it confers upon those who are admitted to architects' fees, was ?2,367, or about ?200 a bed.
treatment. The furniture cost ?725, including fittings for the
The hospital is built, as will be seen from the wards, operating theatre, and dispensary. A further
ground floor plan, upon what is known as the ?600 was spent upon contractors' extras, road-
straight model. It has an extensive verandah look- making, fences, and the mortuary and store-room
ing out on the grounds with a southern aspect. It (?156). The site is 3^ acres in extent, and cost
contains two wards, right and left, which are sepa- ?700. The architects were Messrs. Sharman and
rated from each other by an operation-room, surgery, Archer, of Wellingborough, and we congratulate
bathroom, and a small so-called separation ward, them upon the excellent way in which the work has
which is better suited for a nurse. The lavatories been executed.

				

## Figures and Tables

**Figure f1:**